# Corrosion Prediction with Parallel Finite Element Modeling for Coupled Hygro-Chemo Transport into Concrete under Chloride-Rich Environment

**DOI:** 10.3390/ma10040350

**Published:** 2017-03-28

**Authors:** Okpin Na, Xiao-Chuan Cai, Yunping Xi

**Affiliations:** 1 R&D Division, Hyundai E&C, Yongin-si, Gyeonggi-do 16891, Korea; 2 Computer Science, University of Colorado Boulder, Boulder, CO 80309, USA; xiao-chuan.cai@colorado.edu; 3 Civil, Environmental, and Architectural Engineering, University of Colorado Boulder, Boulder, CO 80309, USA; yunping.xi@colorado.edu

**Keywords:** parallel finite element method, diffusion, coupled hygro-chemo, concrete degradation

## Abstract

The prediction of the chloride-induced corrosion is very important because of the durable life of concrete structure. To simulate more realistic durability performance of concrete structures, complex scientific methods and more accurate material models are needed. In order to predict the robust results of corrosion initiation time and to describe the thin layer from concrete surface to reinforcement, a large number of fine meshes are also used. The purpose of this study is to suggest more realistic physical model regarding coupled hygro-chemo transport and to implement the model with parallel finite element algorithm. Furthermore, microclimate model with environmental humidity and seasonal temperature is adopted. As a result, the prediction model of chloride diffusion under unsaturated condition was developed with parallel algorithms and was applied to the existing bridge to validate the model with multi-boundary condition. As the number of processors increased, the computational time decreased until the number of processors became optimized. Then, the computational time increased because the communication time between the processors increased. The framework of present model can be extended to simulate the multi-species de-icing salts ingress into non-saturated concrete structures in future work.

## 1. Introduction

One of the main long-term durability problems of reinforced concrete structure is the corrosion of reinforcing bars (rebar) in concrete. The corrosion can be resulted from several necessary conditions, and one of them is a high concentration of chloride ions near rebars, where the chloride ions come from deicing chemicals used in the winter on roadways and parking structures or seawater for the off-shore structures. Once the chloride content on the surface of steel reinforcement reaches a threshold value and the moisture and oxygen are sufficiently provided, the corrosion of steel bar is initiated [1,2]. The corrosion initiation period is the time during which substances such as water, chloride ions, oxygen and carbon dioxide penetrate through the concrete cover. The length of period depends on the resistance of concrete to the transport processes and the severity of the environmental conditions they are exposed to [3,4,5,6]. In practical engineering, the chloride contaminated RC structures have many internal cracks due to rebar corrosion and the prediction of crack width and propagation is very important for safety and serviceability [7,8]. Recently, the severity of corrosion damage was demonstrated through the corrosion-induced modeling with a statistical approach on bridge structures [9,10,11]. In concrete bridges, there are various concentrations of moisture and chemicals on the top surface of bridge decks due to the scatter of de-icing chemicals when they are applied. Especially, moisture and chloride concentrations have spatial distributions and seasonal variations. The service life of concrete is affected by a combination of material properties and microclimate. The microclimate is a term for the climatic conditions at the concrete surface or very close to the surface. This condition on the surface has a more decisive effect on the conditions inside the concrete than most other parameters [12]. The model of microclimate such as environmental humidity and temperature was proposed by Bazant et al. [13].

To simulate more realistically the durability performance of reinforced concrete structures, sophisticated numerical methods for fully coupled moisture and chloride transport processes and reliable material models for the transport parameters are needed. In addition, in order to predict accurately the moisture and chloride distributions in a large reinforced concrete structure, a large number of fine finite element meshes are needed. For the use of the large meshes in numerical analysis, in this study, a parallel algorithm will be employed to a parallel processing system with up to 2000 processors. To consider more realistic boundary conditions on concrete structures, humidity model applied with random stochastic process will be demonstrated and approximately 1.5 million nodes and three million elements will be used. Furthermore, multi-boundary conditions to describe the actual chloride concentrations and humidity distribution will be applied instead of only constant boundary conditions.

## 2. Basic Diffusion Formulation of Unsaturated Concrete

### 2.1. Governing Equation

The two fully-coupled partial differential equations governing the coupled chloride and moisture diffusion through non-saturated concrete are simply derived by employing the mass balance equations and Fick’s law [14,15].

First, the flux of chloride ions (*J*) through a unit area of porous media depends on the gradient of chloride ions as well as the gradient of moisture as. Thus, Fick’s law is modified in this study to include the coupling effects between moisture and chloride transfer. The governing equations are shown in Equations (1) and (2).

(1) $$J_{cl}=-(D_{cl}\nabla C_{f}+\varepsilon D_{H}\nabla H)$$

(2) $$J_{H}=-(\delta D_{cl}\nabla C_{f}+D_{H}\nabla H)$$

where *D_cl_* = chloride diffusion coefficient (cm^2^/day); *C_f_* = free chloride concentration (in gram of free chloride per gram of concrete, g/g); *D_H_* = humidity diffusion coefficient; *H* = pore relative humidity; ε = humidity gradient coefficient, which represents the coupling effect of moisture diffusion on chloride penetration; and δ = chloride gradient coefficient, which represents the coupling effect of chloride ions on moisture diffusion.

When chloride ions ingress into the concrete, some of them are bound to the internal surface of the cement paste and aggregates, which are called bound chlorides and freely go through the concrete. Steel corrosion is related only to the free chloride content but not to the total chloride content [14].

The mass balance of chloride ions and moisture can be expressed using Fick’s second law as Equations (3) and (4),

(3) $$\frac{\partial C_{t}}{\partial t}=\frac{\partial C_{t}}{\partial C_{f}}\cdot \frac{\partial C_{f}}{\partial t}=-div(J_{Cl})=div(D_{Cl}\nabla C_{f}+\varepsilon D_{H}\nabla H)$$

(4) $$\frac{\partial w}{\partial t}=\frac{\partial w}{\partial H}\cdot \frac{\partial H}{\partial t}=-div(J_{H})=div(\delta D_{Cl}\nabla C_{f}+D_{H}\nabla H)$$

where

$C_{t}$: total chloride concentration (in gram of free chloride per gram of concrete, g/g);
w: water content;
$\frac{\partial C_{t}}{\partial C_{f}}$: chloride binding capacity; and
$\frac{\partial w}{\partial H}$: moisture binding capacity.

Equations (3) and (4) can be rewritten as,

(5) $$\frac{\partial C_{t}}{\partial C_{f}}\cdot \frac{\partial C_{f}}{\partial t}=\nabla \cdot (D_{Cl}\nabla C_{f}+\varepsilon D_{H}\nabla H)=\nabla \cdot (D_{Cl}\nabla C_{f}+D_{\varepsilon }\nabla H)$$

(6) $$\frac{\partial w}{\partial H}\cdot \frac{\partial H}{\partial t}=div(\delta D_{Cl}\nabla C_{f}+D_{H}\nabla H)=\nabla \cdot (D_{\delta }\nabla C_{f}+D_{H}\nabla H)$$

where $D_{\varepsilon }$ is coupling parameter, $\varepsilon D_{H}$, and $D_{\delta }$ is coupling parameter, $\delta D_{Cl}$.

The general boundary conditions are as below,

(7) $$C_{f}=C_{0}\;\mathrm{on}\;\Gamma_{1}$$

(8) $$D_{cl}\frac{\partial C_{f}}{\partial n}+J_{Cl}+D_{\varepsilon }\frac{\partial H}{\partial n}+\alpha_{cl}(C_{f}-C_{fa})=0\;\mathrm{on}\;\Gamma_{2}$$

(9) $$H=H_{0}\;\mathrm{on}\;\Gamma_{3}$$

(10) $$D_{H}\frac{\partial H}{\partial n}+J_{H}+D_{\delta }\frac{\partial C_{f}}{\partial n}+\alpha_{H}(H-H_{a})=0\;\mathrm{on}\;\Gamma_{4}$$

where $\alpha_{Cl}$ = convective chloride coefficient, $\alpha_{H}$ = convective relative humidity coefficient, $C_{fa}$ = ambient chloride ions, and $H_{a}$ = ambient relative humidity.

$\Gamma_{1}$ and $\Gamma_{3}$ are the part of boundary with constant chloride ions and relative humidity and $\Gamma_{2}$ and $\Gamma_{4}$ are the part of boundary subjected to specified chloride ions and relative humidity flux, respectively. $\Gamma_{1}$ and $\Gamma_{2}$ form the complete boundary surface for the chloride diffusion problem, and $\Gamma_{3}$ and $\Gamma_{4}$ form the moisture diffusion problem [16].

### 2.2. Material Parameters

To numerically solve the chloride diffusion problem, many material parameters must be determined: the moisture capacity (∂w/∂H), chloride binding capacity ($\partial C_{t}/\partial C_{f}$), humidity diffusion coefficient ($D_{H}$), and chloride diffusion coefficient ($D_{Cl}$) (in Equations (5) and (6)) [15].

#### 2.2.1. Moisture Capacity

The moisture capacity of the concrete was developed based on the multiphase and multiscale model [17]. Assuming that the effect of the shrinkage of concrete can be evaluated simply by the average of the moisture capacities of the aggregate and the cement paste as shown in Equation (11),

(11) $$\frac{\partial w}{\partial H}=f_{agg}{\left(\frac{\partial w}{\partial H}\right)}_{agg}+f_{cp}{\left(\frac{\partial w}{\partial H}\right)}_{cp}$$

where $f_{agg}$ and $f_{cp}$ = weight percentages of the aggregate and cement paste, and ${\left(\frac{\partial w}{\partial H}\right)}_{agg}$ and ${\left(\frac{\partial w}{\partial H}\right)}_{cp}$ = moisture capacities of aggregate and cement paste, which can be calculated based on the model developed (Xi et al., 1994a, b) and Xi (1995a, b) [18,19,20,21].

#### 2.2.2. Chloride Binding Capacity

A modified relationship between the bound chloride $C_{b}$, and the free chloride $C_{f}$, was established by Tang and Nilson (1993) based on Freundlich isotherm, and was proposed by Xi and Bazant (1999);

(12) $$C_{b}=\frac{\beta_{C-S-H}}{1000}{\left(\frac{C_{f}}{35.45\beta_{sol}}\right)}^{A}10^{B}$$

which is differentiated with respect to $C_{f}$ and then one would obtain the chloride binding capacity of concrete as defined by Xi and Bazant (1999) to be;

(13) $$\frac{\partial C_{f}}{\partial C_{t}}=\frac{1}{1+\frac{A\cdot 10^{B}\cdot \beta_{C-S-H}}{35450\beta_{sol}}{\left(\frac{C_{f}}{35.45\beta_{sol}}\right)}^{A-1}}$$

where *A* and *B* = chloride adsorption related constants, 0.3788 and 1.14, *β_sol_* = ratio of pore solution volume to concrete weight, L/g, and $\beta_{C-S-H}$ = weight ratio of *C*-*S*-*H* gel to concrete (g/g) [14,22].

Based on the definition of *β_sol_*, the following Equation (14) can be easily derived;

(14) $$\beta_{sol}=\frac{f_{cp}n_{cp}+f_{agg}n_{agg}}{\rho_{sol}}$$

where $f_{cp}$ and $f_{agg}$ = weight percentages of cement paste and aggregate in concrete mix (g/g), $n_{cp}$ = cement paste adsorption isotherm, $n_{agg}$ = aggregate adsorption isotherm, and $\rho_{sol}$ = density of pore solution measured in g/L.

Specific gravities of concrete and *C*-*S*-*H* are similar, therefore, Xi and Bazant (1999) assumed that the weight fraction of *C*-*S*-*H* in concrete, $\beta_{C-S-H}$, is equal to the volume fraction of *C*-*S*-*H* in concrete, $f_{C-S-H}$, then,

(15) $$\beta_{C-S-H}=f_{C-S-H}=\frac{V_{total}-V_{1}-V_{cp}}{V_{total}}=1-f_{1}-f_{cp}$$

where $f_{1}$ = volume fraction of anhydrous pores of cement particles, and $f_{cp}$ = volume fraction of capillary pores of cement paste [14].

#### 2.2.3. Humidity Diffusion Coefficient

The humidity diffusion coefficient of concrete depends on the diffusion coefficients of aggregate and cement paste. Using the composite theory (Christensen, 1979), the effective diffusion coefficient of concrete can be evaluated as following Equation (16),

(16) $$D_{H}=D_{Hcp}\cdot \left(1+g_{i}/\left[\frac{\left(1-g_{i}\right)}{3}+1/\left(\frac{D_{Hagg}}{D_{Hcp}}-1\right)\right]\right)$$

where $g_{i}$ = aggregate volume fraction, $D_{Hcp}$ = humidity diffusion coefficient of the cement paste, and $D_{Hagg}$ = humidity diffusion coefficient of the aggregates [23].

The humidity diffusion coefficient of aggregates in concrete is very small due to the fact that the pores in aggregates are discontinuous and enveloped by cement paste and so it can be neglected. The humidity diffusion coefficient of cement paste can be predicted by using the empirical formula described in Xi et al. (1994b) [19].

#### 2.2.4. Chloride Diffusion Coefficient

Chloride diffusion coefficient in saturated concrete was studied by Xi and Bazant (1999) as following Equation (17),

(17) $$D_{cl}=f_{1}(w/c,t_{0})\cdot f_{2}(g_{i})\cdot f_{3}(H)\cdot f_{4}(T)\cdot f_{5}(C_{f})$$

In Equation (17), the functions of the chloride diffusivity consist of the concrete curing time ($t_{0}$), gravel volume fraction ($g_{i}$), relative humidity (*H*), temperature (*T*), and free chloride concentration ($C_{f}$) [14].

First factor of chloride diffusion coefficient accounts for the effect of the water–cement ratio (w/c) and curing time ($t_{0}$) in Equation (18),

(18) $$f_{1}(w/c,t_{0})=\frac{28-t_{0}}{62500}+\left(\frac{1}{4}+\frac{\left(28-t_{0}\right)}{300}\right)(w/c{)}^{6.55}$$

The second influence factor is to consider the effect of composite action of the aggregate and the cement paste in Equation (19),

(19) $$f_{2}(g_{i})=D_{cp}\left(1+\frac{g_{i}}{\left[1-g_{i}]/3+1/[(D_{agg}/D_{cp})-1\right]}\right)$$

where $g_{i}$ = volume fraction of aggregate in concrete, and $D_{agg}$ and $D_{cp}$ = chloride diffusion coefficient of aggregate and cement paste.

The third factor, *f*_3_(*H*), in Equation (20) is to consider the effect of relative humidity level on the chloride diffusion coefficient. A model proposed by Bazant et al. (1972) can be used, which was developed initially for moisture diffusion [24].

(20) $$f_{3}(H)={\left[1+{\left(\frac{1-H}{1-H_{C}}\right)}^{4}\right]}^{-1}$$

where Hc = critical humidity level, 0.75.

Arrhenius law was used by Xi and Bazant (1999) to introduce the temperature effect of the forth factor in chloride diffusion coefficient as shown in Equation (21),

(21) $$f_{4}(T)=\exp\left[\frac{U}{R}\left(\frac{1}{T_{0}}-\frac{1}{T}\right)\right]$$

where

$T_{0}$ and T = reference and current temperatures in Kelvin, *T*_0_ = 296 K;
*R* = gas constant, 8.314 J/mol·K; and
*U* = diffusion process activation energy, depending on *w*/*c* ratio [14].

The detail description of diffusion process activation energy, *U* can be found in the paper of Ababneh et al. (2003) [15].

The dependence of chloride diffusion coefficient on free chloride concentration $C_{f}$ for the fifth factor is presented in Equation (22);

(22) $$f_{5}(C_{f})=1-k_{ion}{\left(C_{f}\right)}^{m}$$

where $k_{ion}$ and *m* = 8.333 and 0.5, respectively, according to Xi and Bazant (1999) [14].

## 3. Finite Element Formulation

In order to solve a time-dependent coupled moisture-chloride diffusion problem, a large linear system equation was derived with finite element method. The finite element formulation will be briefly introduced in this section. The continuous variables in the coupled chloride and moisture diffusion equations, free chloride ($C_{f}$) and relative humidity ($H_{m}$) are spatially discretized over the space domain, Ω. The domain discretization can be described as shown in Equation (23).

(23) $$\Omega =\overset{nel}{\underset{e=1}{\cup }}\Omega^{e}$$

in which *nel* is the total number of elements in space domain and $\Omega_{e}$ is an element. It is also defined ∂Ω as the boundary of computational domain and $\partial \Omega^{e}$ the boundary of subdomain.

The unknown variables in Equations (24) and (25) are defined in terms of nodal values, $\left\{C_{f}\right\}$ and $\left\{H_{m}\right\}$,

(24) $$C_{f}≃\left\lfloor N\right\rfloor \left\{{\overset{\wedge }{C}}_{f}\right\}$$

(25) $$H_{m}≃\left\lfloor N\right\rfloor \left\{{\overset{\wedge }{H}}_{m}\right\}$$

where ⌊N⌋ is the triangle element shape function. The notations ⌊⌋ and {} are row and column vectors, respectively. The element shape functions are expressed as following Equation (26),

(26) $$\left\lfloor N\right\rfloor =\left\lfloor N_{1}\;N_{2}...\;N_{n}\right\rfloor$$

in which *N_i_* is the shape function for node *i* and *n* is the total numbers of nodes in an element. The unknown vectors of free chloride $\left\{\overset{\wedge }{C_{f}}\right\}$ in Equation (27) and relative humidity $\left\{\overset{\wedge }{H_{m}}\right\}$ in Equation (28) can be defined as,

(27) $$\left\{{\overset{\wedge }{C}}_{f}\}\equiv \{{\overset{\wedge }{C}}_{1},{\overset{\wedge }{C}}_{2},{\overset{\wedge }{C}}_{3},\cdot \cdot \cdot \cdot \cdot \cdot \cdot \cdot \cdot \cdot \cdot ,{\overset{\wedge }{C}}_{n}\right\}$$

(28) $$\left\{{\overset{\wedge }{H}}_{m}\}\equiv \{{\overset{\wedge }{H}}_{1},{\overset{\wedge }{H}}_{2},{\overset{\wedge }{H}}_{3},\cdot \cdot \cdot \cdot \cdot \cdot \cdot \cdot \cdot ,{\overset{\wedge }{H}}_{n}\right\}$$

The nodal free chloride concentrations and relative humidity are solved by substituting the approximated values of Equations (24) and (25) into governing equations of Equations (5) and (6), and applying the Galerkin procedure to the weak forms, then the finite element matrix can be obtained as shown in Equation (29):

(29) $$\frac{d}{dt}\left(\left[C_{e}(\overset{\wedge }{\phi })\right]\left\{\overset{\wedge }{\phi }\right\}\right)=\left[K_{e}(\overset{\wedge }{\phi })\right]\left\{\overset{\wedge }{\phi }\right\}$$

where the element matrices and vector are as following Equations (30)–(32),

(30) $$\left[C_{e}\right]=\left[\begin{array}{cc} C_{c} & 0\\ 0 & C_{h} \end{array}\right]$$

(31) $$\left[K_{e}\right]=\left[\begin{array}{cc} K_{cc} & K_{ch}\\ K_{hc} & K_{hh} \end{array}\right]$$

(32) $$\{\overset{\wedge }{\phi }\}=\left\lfloor {\overset{\wedge }{C}}_{f}\overset{\wedge }{H_{m}}\right\rfloor$$

In detail, the components in element matrices are as Equations (33)–(38),

(33) $$\left[K_{cc}\right]=-\int_{\Omega_{e}}\nabla {\left\lfloor N_{c}\right\rfloor }^{T}D_{Cf}\nabla \left\lfloor N_{c}\right\rfloor d\Omega +\int_{\partial \Omega_{e}}{\left\lfloor N_{c}\right\rfloor }^{T}D_{Cf}\nabla \left\lfloor N_{c}\right\rfloor d\Gamma$$

(34) $$\left[K_{ch}\right]=-\int_{\Omega_{e}}\nabla {\left\lfloor N_{c}\right\rfloor }^{T}D_{\varepsilon }\nabla \left\lfloor N_{h}\right\rfloor d\Omega +\int_{\partial \Omega_{e}}{\left\lfloor N_{c}\right\rfloor }^{T}D_{\varepsilon }\nabla \left\lfloor N_{h}\right\rfloor d\Gamma$$

(35) $$\left[K_{hc}\right]=-\int_{\Omega_{e}}\nabla {\left\lfloor N_{h}\right\rfloor }^{T}D_{\delta }\nabla \left\lfloor N_{c}\right\rfloor d\Omega +\int_{\partial \Omega_{e}}{\left\lfloor N_{h}\right\rfloor }^{T}D_{\delta }\nabla \left\lfloor N_{c}\right\rfloor d\Gamma$$

(36) $$\left[K_{hh}\right]=-\int_{\Omega_{e}}\nabla {\left\lfloor N_{h}\right\rfloor }^{T}D_{Hm}\nabla \left\lfloor N_{h}\right\rfloor d\Omega +\int_{\partial \Omega_{e}}{\left\lfloor N_{h}\right\rfloor }^{T}D_{Hm}\nabla \left\lfloor N_{h}\right\rfloor d\Gamma$$

(37) $$\left[C_{C}\right]=\int_{\Omega_{e}}{\left\lfloor N_{C}\right\rfloor }^{T}C_{C}\left\lfloor N_{C}\right\rfloor d\Omega$$

(38) $$\left[C_{h}\right]=\int_{\Omega_{e}}{\left\lfloor N_{h}\right\rfloor }^{T}C_{h}\left\lfloor N_{h}\right\rfloor d\Omega$$

Finally, Equation (39) is also discretized in time space with time interval $△t=t^{\xi +1}-t^{\xi }$ as following,

(39) $${\left(\left[C_{e}(\overset{\wedge }{\phi })-\theta \cdot \Delta t\cdot K_{e}(\overset{\wedge }{\phi })\right]\left\{\overset{\wedge }{\phi }\right\}\right)}^{\xi +1}={\left(\left[C_{e}(\overset{\wedge }{\phi })-(1-\theta )\cdot \Delta t\cdot K_{e}(\overset{\wedge }{\phi })\right]\left\{\overset{\wedge }{\phi }\right\}\right)}^{\xi }$$

The value of parameter θ is related to the solution method adopted in the program. Typical values of θ are 0, 1/2 and 1 correspond to fully explicit, semi-implicit and fully implicit methods, respectively. The semi-implicit method called Crank–Nicholson method is used in this study.

Equation (39) is simplified as linear system equation.

(40) $${\left[A\right]}^{\xi +1}{\left\{\overset{\wedge }{\phi }\right\}}^{\xi +1}={\left\{b\right\}}^{\xi }$$

where

(41) $${\left[A\right]}^{\xi +1}={\left[C_{e}(\overset{\wedge }{\phi })-\theta \cdot \Delta t\cdot K_{e}(\overset{\wedge }{\phi })\right]}^{\xi +1}$$

(42) $${\left\{b\right\}}^{\xi }={\left(\left[C_{e}(\overset{\wedge }{\phi })-(1-\theta )\cdot \Delta t\cdot K_{e}(\overset{\wedge }{\phi })\right]\left\{\overset{\wedge }{\phi }\right\}\right)}^{\xi }$$

## 4. Implementation of Parallel Finite Element Method

### 4.1. Various Programs Adapted in Parallel Finite Element Program

In order to implement the parallel finite element program for the coupled moisture and chloride problem, various programs were employed such as Triangle for mesh generation, ParMETIS, PETSc (Portable, Extensible Toolkit for Scientific Computation), and MPI (Message Passing Interface) [25,26,27,28,29,30,31].

In this study, Triangle was used for the mesh generation of triangle element, which was created at Carnegie Mellon University. Triangle generates exact delaunay triangulations, and are suitable for finite element analysis [25].

PETSc (3.0.0 p8) is a large and versatile package integrating distributed vectors, distributed matrices in several sparse storage formats, Krylov subspace methods, preconditioners, and Newton-like nonlinear methods with built-in trust region or linesearch strategies and continuation for robustness. It is designed to provide the numerical infrastructure for application codes involving the implicit numerical solution of PDEs, and it uses on MPI for portability to most parallel machines. The PETSc library is written in C, but may be accessed from user codes written in C, Fortran, and C++ [32].

MPI (Message Passing Interface) is a standardized and portable message-passing system designed to function on a wide variety of parallel computers. The standard defines the syntax and semantics of library routines and allows users to write portable programs in the main scientific programming languages (Fortran, C, or C++) [27,28,29].

ParMETIS extends the functionality of METIS and includes routines based on a parallel graph-partitioning algorithm that are especially suited for parallel computations and large-scale numerical simulations involving unstructured meshes. In typical FEM computations, ParMETIS dramatically reduces the time spent in interprocess communication by computing mesh decompositions such that the number of interface nodes/elements is minimized [30].

### 4.2. Overlapping Domain Decomposition Method with Additive Schwarz Preconditioner

A parallel program is typically developed by dividing the program into multiple fragments that can execute simultaneously, each on its own processor. In the finite element analysis, this can be accomplished by applying a domain decomposition method. Domain decomposition method is the method usually used for solving large scale system equations and it is also suitable for parallel programming because of data locality [15]. There are two types of domain decomposition methods, overlapping and non-overlapping methods. In this study, the overlapping method was employed to solve the linear sparse matrix. That is because the advantage of overlapping domain decomposition is easier to setup in algebraic approach and faster convergence than non-overlapping domain decomposition. Furthermore, the boundaries of extended subdomains are smoother than non-overlapping subdomains.

**Figure 1:**
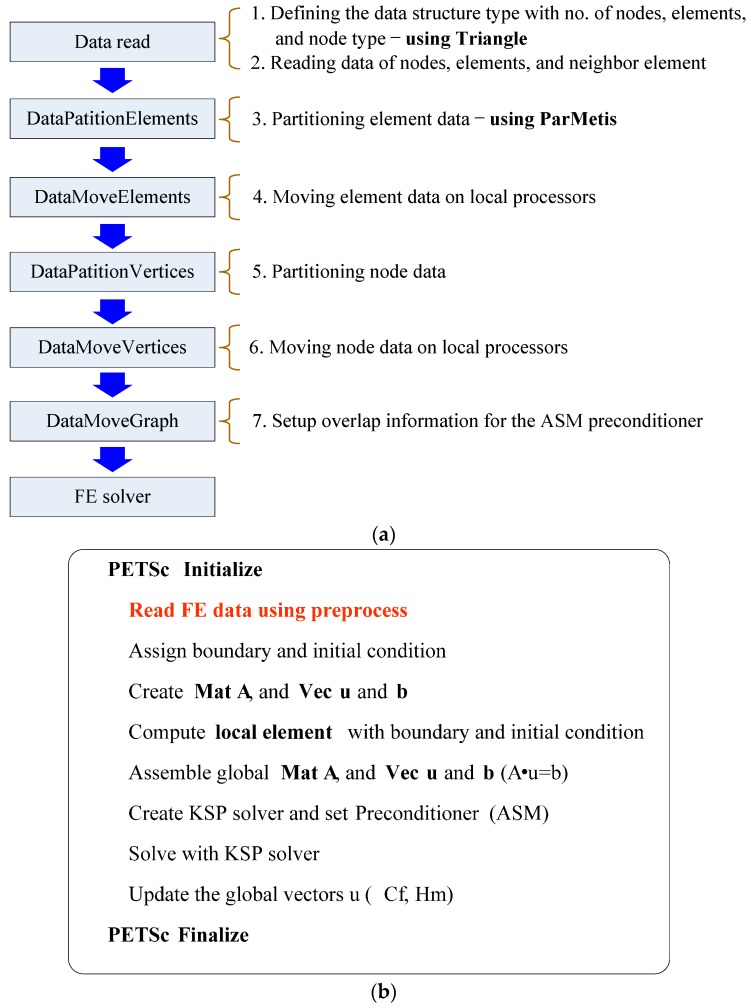
Framework of parallel FE method based on PETSc: (**a**) flow chart for parallel pre-process; and (**b**) flow chart for parallel FE solver.

Iterative solver must be used in the iterative domain decomposition method. In this study, for the iterative solver, GMRES (Generalized Minimal Residual method) was chosen for both global and local matrix. GMRES is mainly chosen because of its ability to solve non-symmetric linear system as in the case of our problem. To improve the convergence of this problem, the additive Schwarz method preconditioner was applied. Figure 1 shows the flow chart of parallel pre-process and FE solver.

## 5. Numerical Results

### 5.1. Applied Bridge Overview

**Figure 2:**
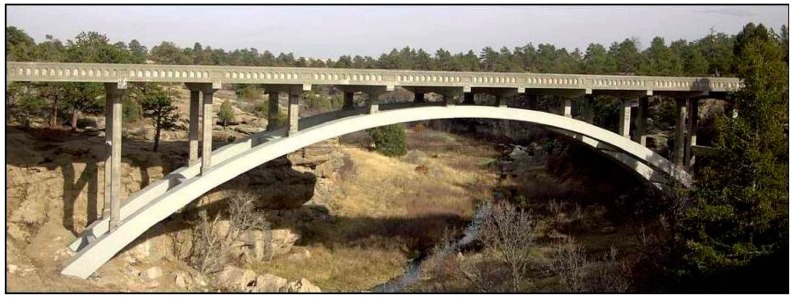
Castlewood Canyon bridge, Franktown, Colorado.

The Castlewood Canyon bridge is located on Highway 83 in the Black Forest of central Colorado. The original two-lane reinforced concrete arch bridge was built in 1946. The arches are 1.93 m wide by 1.78 m deep at the base with the depth tapering down to 1.0 m at the highest point. This bridge was severely dilapidated and was in need of repairing, enlarging, and strengthening. Parts of the concrete had spalled off of the deck, columns, and arches, and the steel rebar under concrete were severely rusted. In 2003, the original arch was repaired, the spandrel columns and decks were replaced and widened from about 0.9 m to 1.0 m including railings, and the overall length of the bridge was increased from 114 m to 123 m as shown in Figure 2.

### 5.2. Parallel Finite Method of Large-Scale Concrete Structure

#### 5.2.1. Modeling of Castle Wood Canyon Bridge with Large Meshes

In order to analyze the penetration of chloride and humidity in a concrete bridge, a large number of meshes are needed to capture the diffusion phenomenon within thin layer from concrete top surface to steel rebar. For depicting in detail concrete cover depth on rebar, the information of nodes and elements are created by mesh generator. Triangle as mesh generator is specialized for creating two-dimensional finite element meshes.

**Figure 3:**
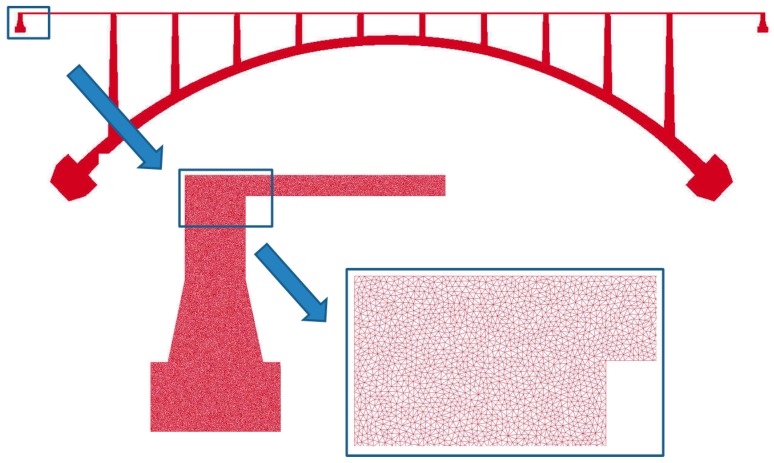
Modelling of Castlewood Canyon bridge.

**Figure 4:**
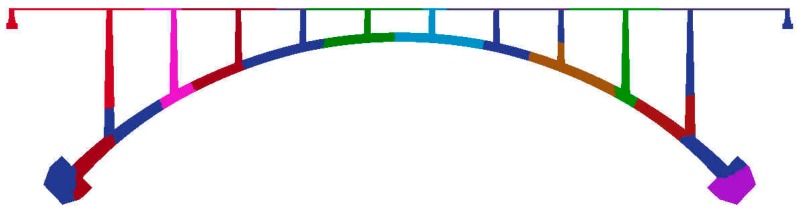
Partitioning of Castlewood Canyon bridge with 16 processors.

For castle wood canyon bridge, approximately 1.5 million nodes and three million elements were generated for input files as shown in Figure 3. To visualize and check the mesh size and shape, ParaView was employed as illustrated in Figure 4 [33]. ParaView is an open-source, multi-platform data analysis and visualization application. It is developed to analyze extremely large datasets using distributed memory computing resources and can be run on supercomputers to analyze datasets of terascale as well as on laptops for smaller data. For large scale information, VTK file format is adopted because this format is easy to read and write by hand or programmatically. This file format is automatically created when the program runs. For partitioning origin meshes, Parmetis was used to be embedded in the parallel program to automatically partition. Parmetis is an MPI-based parallel library that implements a variety of algorithms for partitioning unstructured graphs, meshes, and for computing fill-reducing orderings of sparse matrices. ParMETIS extends the functionality provided by METIS and includes routines that are especially suited for parallel AMR computations and large scale numerical simulations. The algorithms implemented in ParMETIS are based on the parallel multilevel k-way graph-partitioning, adaptive repartitioning, and parallel multi-constrained partitioning schemes developed.

#### 5.2.2. Environmental Humidity Model

**Figure 5:**
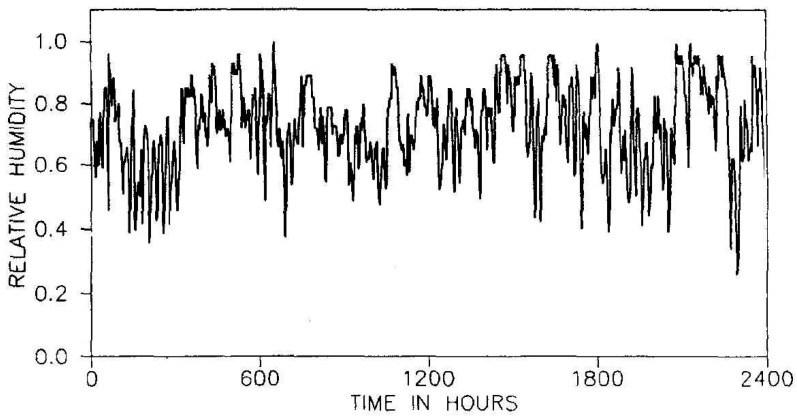
Environmental humidity Record, midway station, Chicago (Bazant et al., 1993) [13].

Environmental humidity model plays an important role of diffusion of chloride in concrete. The humidity model used in this study was proposed by Bazant and Xi (1993) [13]. Even though the moisture and temperature transport in concrete were coupled, humidity fluctuation was considered and temperature was assumed to be constant during analysis time. To establish a practicable and realistic humidity input model, real climate record in Chicago was employed as shown in Figure 5.

**Figure 6:**
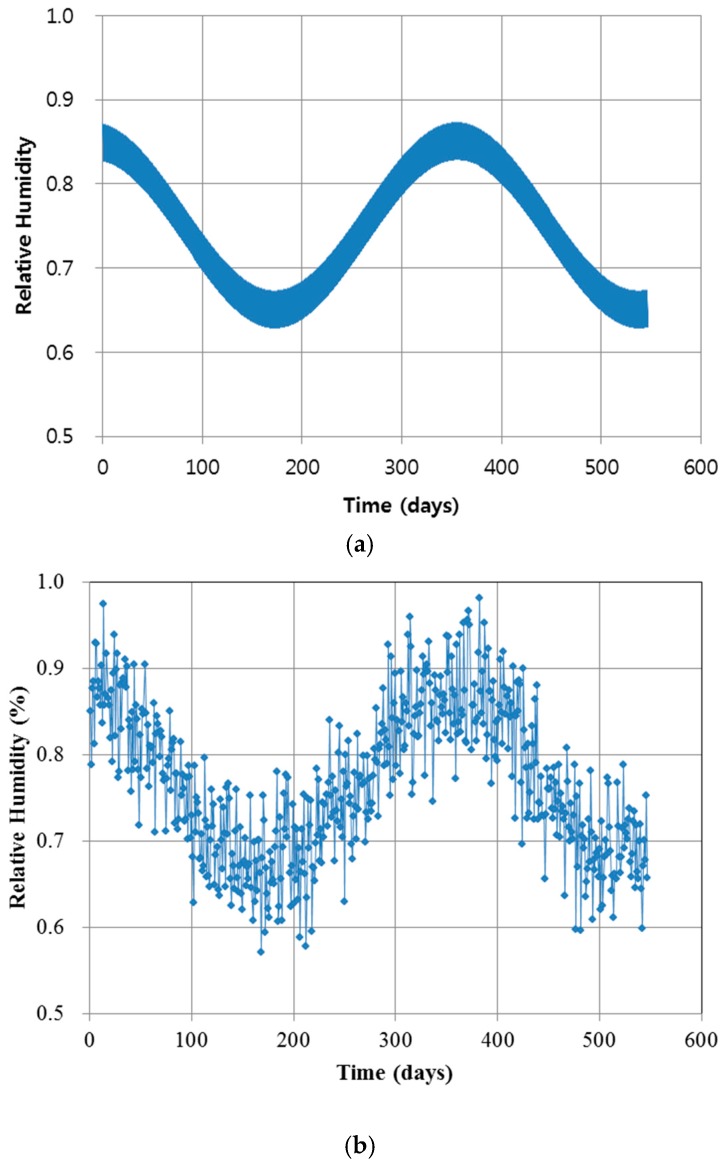
Environmental humidity model: (**a**) humidity model without random noise; and (**b**) humidity model with three components (including random noise).

The environmental humidity model consists of three different components as shown in Equation (43) and demonstrated in Figure 6. First, $H_{1}$ is the mean value, standing for the stable horizontal trend. Second, $H_{2}$ is a random phase process corresponding to the harmonic variation of humidity. Third, $H_{3}$ is a random normal distribution with a specific mean and variance [13].

(43) $$H=H_{1}+H_{2}+H_{3}$$

where $H_{1}=\bar{H}$, mean value of environmental model, $H_{2}=A_{1}\;\cos(2\pi \;t\;+\varphi_{1})+\;A_{365}\;\cos(2\pi \;t\;+\varphi_{365})$, the random phase process of a one day period with $A_{1}$ = 0.1 and the random phase process of a one year period with $A_{365}$ = 0.08, $\varphi_{1}$ and $\varphi_{365}$ have uniform distributions with constant density 12π, $H_{3}=\frac{1}{\sqrt{2\pi \sigma^{2}}}e^{\left[-\frac{{\left(x-\mu \right)}^{2}}{2\sigma^{2}}\right]}$, normally distributed random numbers are generated with specific mean and variance, 0.0046 [13].

**Figure 7:**
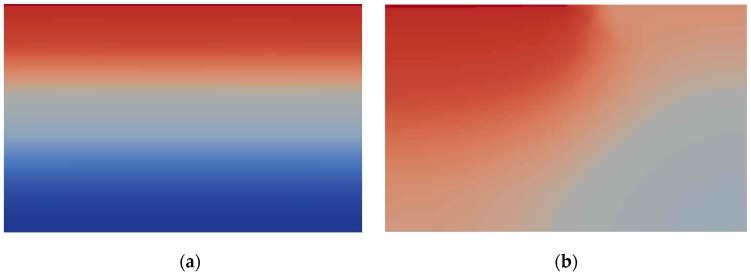
Comparison of single and various boundary conditions: (**a**) single boundary condition; and (**b**) two boundary conditions.

For the chloride concentration on the top surface of concrete, various boundary conditions were used to actualize the realistic boundary conditions as illustrated in Figure 7. One can see that the interference between the interfaces of different concentration.

#### 5.2.3. Speed-Up for Parallel Algorithm

Speed-up is measured for the performance of the parallel implementation of finite element program. The definition of speed-up is a process for increasing in performance between two systems processing the same problem. With regard to speed-up in this study, 8–2048 processors were used in this study and the meshes with the number of about 1.5 million nodes were analyzed. When using up to eight processors, the program was not operated due to memory capacity when input data can be assigned on each memory which means the problem was too huge to solve with a couple of processors.

**Figure 8:**
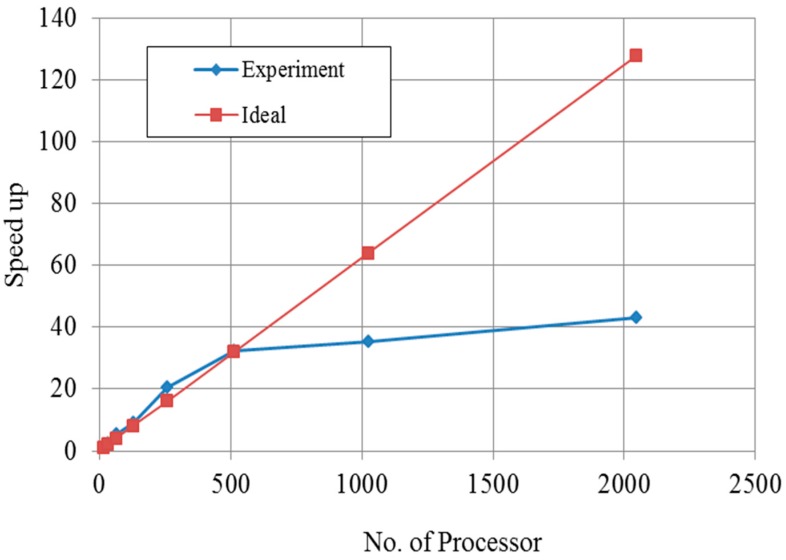
Speed up over number of processors.

As the number of processors used in the analysis increased, speed-up was improved up to 512 processors because speed-up was better than ideal condition. After more than 512 processors were used, speed-up gradually increased because the communication time between the processors increased. As shown in Figure 8, one can see the optimum point of number of processors.

#### 5.2.4. Effect of Boundary Condition

(1) Constant boundary condition

The physical and material models of the castle wood canyon bridge used in this paper were mentioned above. The validation of these models was secured by Ababneh et al. and Suwito et al. [14,15]. Ababneh et al. proved the model with alternating-direction implicit (ADI) finite-difference method and the numerical solutions were compared with the experimental results obtained by the 90-day ponding test (AASHTO T 259-80) [14]. Suwito et al. verified the accuracy of the model implemented with finite element method and numerical results agreed well with the experimental data [15].

It was assumed that the concrete bridge contained initially no chloride ions and has 60% relative humidity (RH). The top surface of concrete bridge was exposed to 3% NaCl and 100% RH. To analyze the diffusion of coupled chloride and humidity, 8 to 2048 processors and approximately 1.5 million nodes were used with parallel finite element method.

Obviously, this example is not to simulate the concrete behavior under service condition. The triangle element meshes were employed and the whole domain was partitioned into 16 sub-domains to the same as the number of processors shown in Figure 3 and Figure 4. The sub-domains were divided with ParMetis so that each sub-mesh had almost the same number of elements.

**Figure 9:**
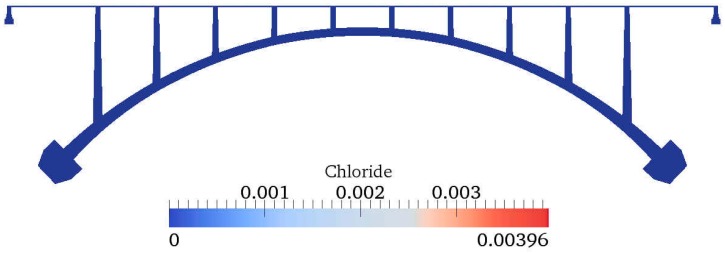
Contour of chloride distribution.

**Figure 10:**
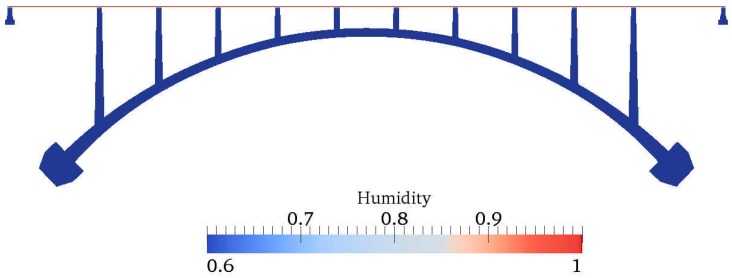
Contour of humidity distribution.

**Figure 11:**
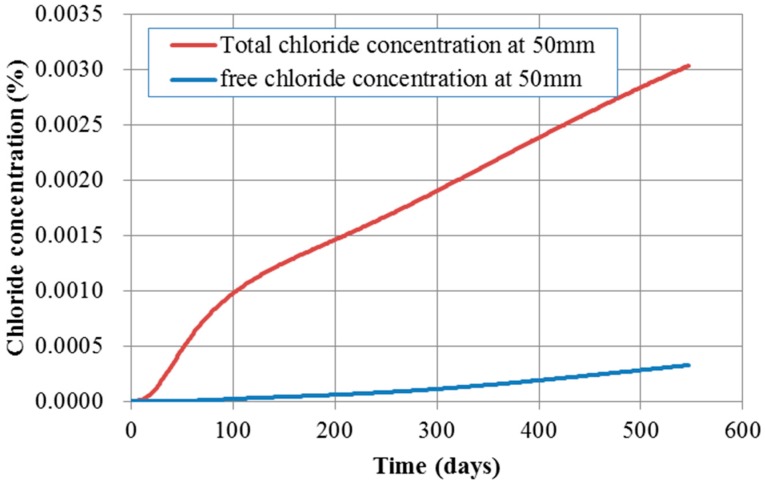
Chloride concentration over time at 50 mm depth.

**Figure 12:**
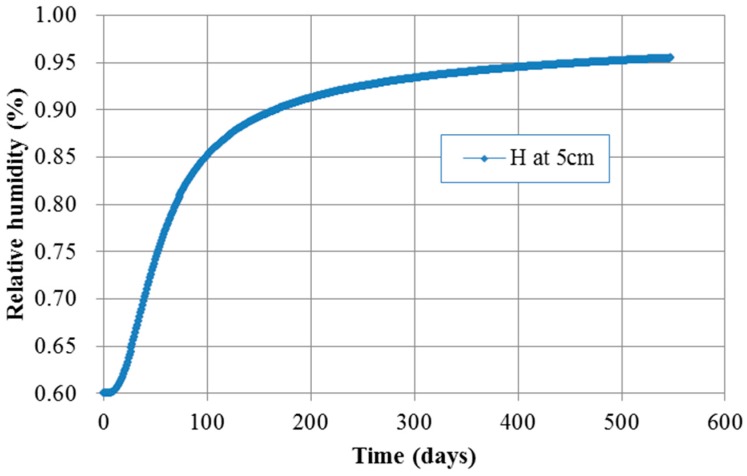
Humidity over time at 50 mm depth.

Figure 9 and Figure 10 show the contour of chloride and humidity distribution into the entire domain. The total and free chloride concentration at 50 mm below from the top surface of concrete is Figure 11 and the variation of relative humidity is Figure 12 over time up to 550 days. As a result of analysis, the concentration of chloride and humidity is continuously accumulated inside concrete. Specially, the variation of humidity inside concrete might not be increased in reality due to the seasonally change of the humidity distribution, so that periodic humidity model should be applied as the boundary condition.

(2) Periodic boundary condition

**Figure 13:**
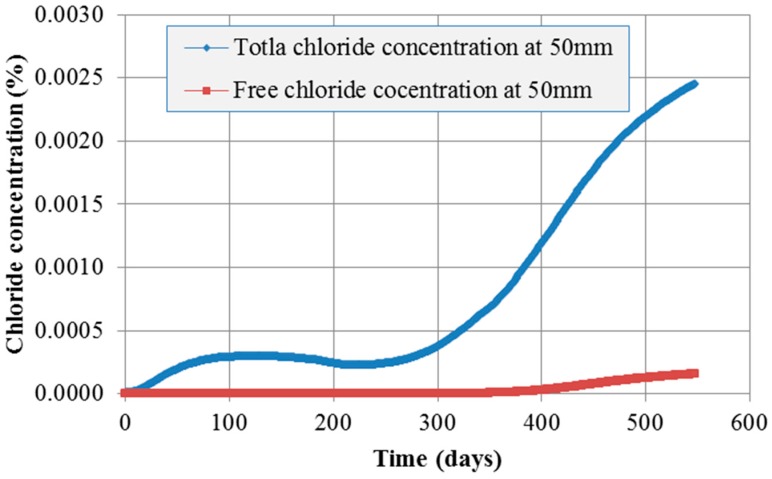
Chloride concentration over time at 50 mm depth.

**Figure 14:**
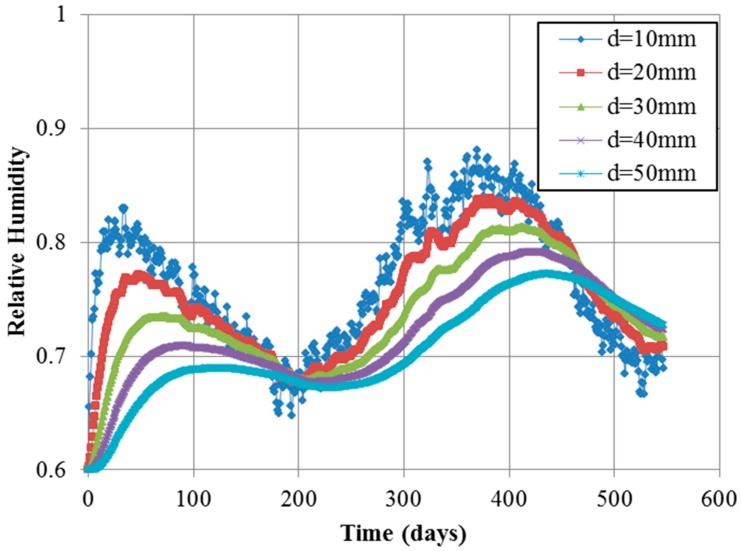
Humidity over time according to depth.

In order to simulate the real diffusion phenomenon in concrete, the periodic boundary condition should be required. Figure 13 and Figure 14 describe the result of chloride concentration and relative humidity at different depth over time. As shown in Figure 13, total concentration is gradually increasing until almost one year. That means the chloride concentration can be affected by the periodic humidity boundary condition. After one year it suddenly increases because of the influence of the constant chloride initial condition. However, the total chloride concentration at 50 mm is reduced up to 15% rather than the concentration using constant boundary. In order to predict and demonstrate the realistic and seasonal chloride ingress through concrete cover, seasonal change information of chloride concentration on the top surface of concrete deck.

In Figure 13, the change of humidity distribution is influenced from the humidity model inputted as boundary condition. The short period random noise, one of random noise terms in environmental humidity model, can only reach a shallow portion near the top surface of concrete deck. As the concrete depth increased, the effect of short period random noise decreases. However, the seasonal variation of humidity concentration has an influence on 50 mm depth of concrete deck. At 50 mm from the top surface of concrete deck, the maximum value of humidity is about 77%, which is decreased up to 20% comparing with constant boundary condition. In addition, the result of about 500 days shows that the humidity trend is reversed between 10 mm and 50 mm, which means that evaporation of humidity just near concrete surface is easier than evaporation at 50 mm. Therefore, this prediction model is reasonable and effective to describe the diffusion phenomenon of chloride and humidity.

## 6. Conclusions

(1) The parallel finite element program was developed based on the robust mathematical material model. The program can be used to simulate the coupled moisture and chloride penetration into non-saturated concrete structures. Chloride ion is one of sources causing the steel corrosion in reinforced concrete structures. The material parameters related to chloride and moisture diffusion in concrete are taken into account. These parameters include chloride and moisture diffusion coefficients, moisture capacity, chloride binding capacity, and the coupling parameters reflecting the coupling effects between moisture and chloride transfer in concrete.
(2) For the implementation of parallel FE analysis, Triangle for mesh generation, ParMetis, PETSc, and MPI are employed. This program also used the overlapping domain decomposition method with additive Schwarz preconditioner. As the result of simulation, the computation time decreased until the number of processors became optimized when the number of processors increased. Then, the computational time increased because the communication between the processors increased.
(3) The present model can be used to simulate the unsaturated concrete structures subjected to other aggressive chemicals from de-icing salt. The framework of present model can be extended to simulate the multi-species de-icing salts ingress into non-saturated concrete structures in future work.
